# Nanomaterials‐assisted metabolic analysis toward in vitro diagnostics

**DOI:** 10.1002/EXP.20210222

**Published:** 2022-04-16

**Authors:** Jing Yang, Lin Huang, Kun Qian

**Affiliations:** ^1^ State Key Laboratory for Oncogenes and Related Genes, School of Biomedical Engineering, Institute of Medical Robotics and Med‐X Research Institute Shanghai Jiao Tong University Shanghai China; ^2^ Department of Obstetrics and Gynecology, Renji Hospital, School of Medicine Shanghai Jiao Tong University Shanghai China; ^3^ Country Department of Clinical Laboratory Medicine Shanghai Chest Hospital Shanghai Jiao Tong University Shanghai China

**Keywords:** biomarkers, fingerprints, in vitro diagnostics, metabolite, nanomaterials

## Abstract

In vitro diagnostics (IVD) has played an indispensable role in healthcare system by providing necessary information to indicate disease condition and guide therapeutic decision. Metabolic analysis can be the primary choice to facilitate the IVD since it characterizes the downstream metabolites and offers real‐time feedback of the human body. Nanomaterials with well‐designed composition and nanostructure have been developed for the construction of high‐performance detection platforms toward metabolic analysis. Herein, we summarize the recent progress of nanomaterials‐assisted metabolic analysis and the related applications in IVD. We first introduce the important role that nanomaterials play in metabolic analysis when coupled with different detection platforms, including electrochemical sensors, optical spectrometry, and mass spectrometry. We further highlight the nanomaterials‐assisted metabolic analysis toward IVD applications, from the perspectives of both the targeted biomarker quantitation and untargeted fingerprint extraction. This review provides fundamental insights into the function of nanomaterials in metabolic analysis, thus facilitating the design of next‐generation diagnostic devices in clinical practice.

## Graphical abstract

Nanomaterials have found extensive applications in the metabolic analysis of diverse bio‐specimens, with efficient selectivity and sensitivity. The nanomaterial‐based metabolic analysis is always accompanied by accurate quantitation of targeted metabolite biomarkers and high‐throughput profiling of untargeted metabolic fingerprints, adding practical value to in vitro diagnostics. Its further developments will have far‐reaching implications for the research of precision medicines.

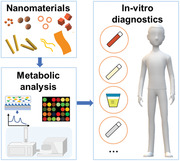

## 1 INTRODUCTION

In vitro diagnostics (IVD) has played an indispensable role in healthcare system by providing necessary information to indicate disease condition and guide therapeutic decisions.^[^ ^1^, ^2^, ^3^ ^]^ Distinguishing from in vivo diagnostics that perform directly on the body, IVD can be conducted on the biological samples of tissue and biofluids (e.g., serum, urine, and saliva).^[^ ^4^, ^5^ ^]^ Notably, IVD has been the focus of interdisciplinary innovations and contributed to a market share of more than 70% of clinical diagnostics due to its prominent features: (1) it offers limited invasiveness, which can improve patient compliance and provide the feasibility of continuous monitoring; (2) it measures molecular or cellular features of biospecimens, avoiding the disturbance of body condition raised by invasive equipment; and (3) it requires simple devices only, displaying preferable adaptability to point‐of‐care testing (POCT) especially in regions with limited resources. One of the most popular examples in IVD is diabetic glucose meters,^[^ ^6^ ^]^ the simplification of which significantly improved daily management of diabetes.

To further achieve early‐stage diagnosis and accurate subtyping of diseases, two key factors in IVD systems are required to be addressed, including deciding suitable targets with in‐time feedback and designing device integration with high performance. For targets, metabolites, that is, small molecules with molecular weight of <1000 Da, are considered the desirable biomarkers for IVD.^[^ ^7^, ^8^, ^9^ ^]^ Specifically, metabolites provide a distal characterization of the human body by reflecting upstream nucleic acids and proteins and indicating downstream metabolism dynamics. Nowadays, the analysis of targeted metabolite biomarkers has become a vital constituent of IVD.^[^ ^10^, ^11^ ^]^ For instance, creatinine is a functional metabolite biomarker and enjoys a broad application in clinical practice, since its level in serum reflects renal conditions.^[^ ^12^, ^13^ ^]^

For device integration, advances in high‐throughput detection technologies place the metabolic analysis at the forefront of precise medicine, as it decodes the disease mechanism with newly discovered metabolite biomarkers.^[^ ^14^, ^15^ ^]^ Recently, nanomaterials with designed compositions and nanostructures were developed and integrated into diverse detection platforms for metabolic analysis with enhanced performance.^[^ ^16^, ^17^ ^]^ For example, quantum dots (QDs)^[^ ^18^, ^19^ ^]^ and plasmonic nanoshells^[^ ^20^, ^21^, ^22^ ^]^ can be exploited to enhance the metabolic signal due to their unique optical properties and plasmonic resonance effect. Taking nanotubes/nanorods as another example, the nanomaterials offer a large surface‐to‐volume ratio and high electrical conductivity, potentially amplifying metabolite signals via the elevated electron transfer.^[^ ^17^, ^23^, ^25^ ^]^ In addition, nanosheets exhibit a large specific surface area, thereby increasing adsorption sites for small molecule metabolites.^[^ ^26^, ^27^, ^28^, ^29^, ^30^, ^31^, ^32^, ^33^ ^]^ Briefly, the rational design of nanomaterials opens a new chapter of IVD, via assisting metabolic detection platforms with enhanced sensitivity and analytical capacity.

**FIGURE 1:**
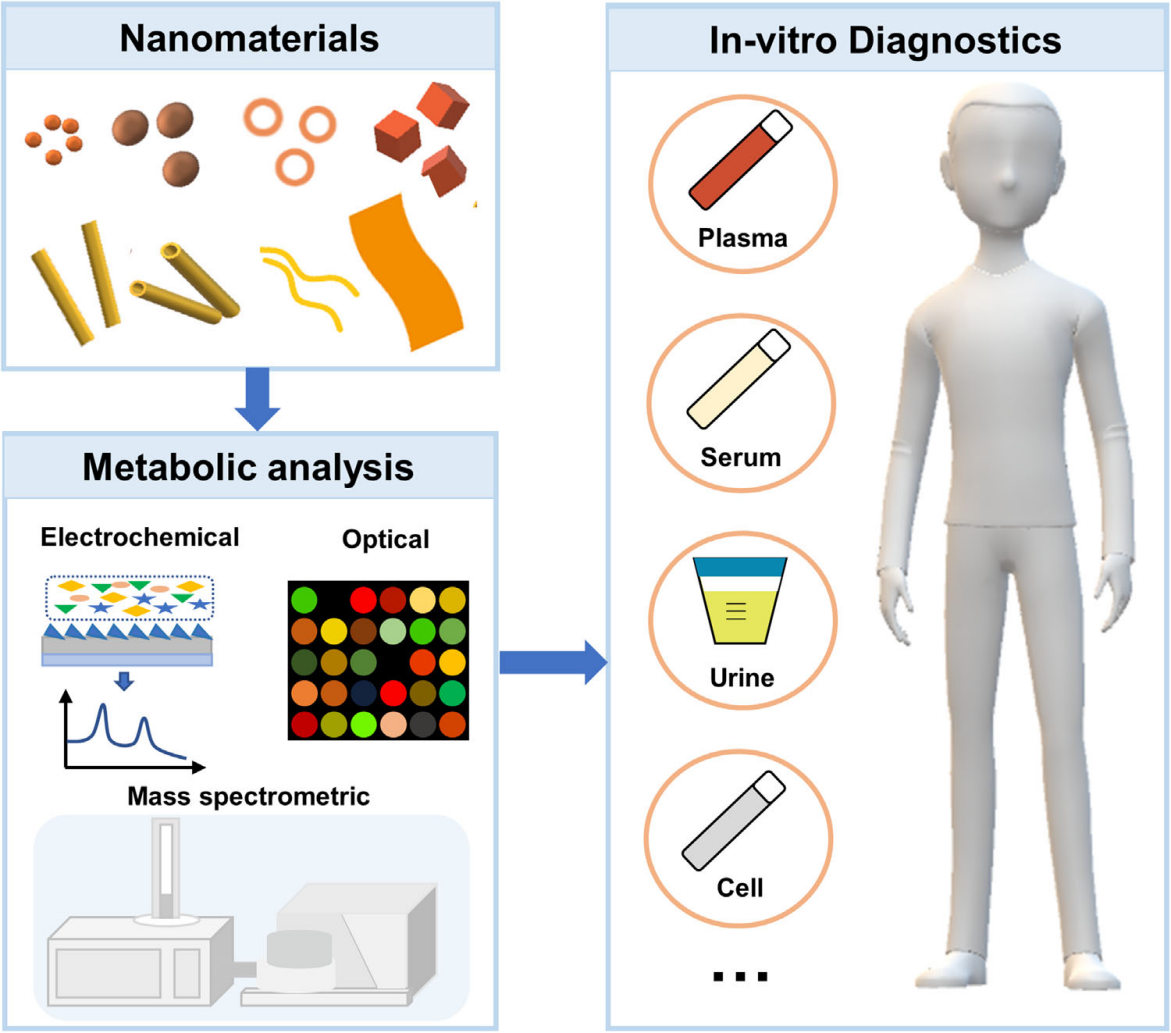
Schematics of functions and applications of nanomaterials in in‐ vitro diagnostics (IVD). We summarize the roles that nanomaterials play in metabolic analysis when coupled with different analytical approaches, including electrochemical, optical, and mass spectrometric (MS) tools. With regard to IVD applications, we discuss the recent advances in the integration of nanomaterials with analytical techniques toward the targeted quantitation of metabolic biomarkers and untargeted fingerprinting of metabolic signatures

Herein, we summarize and describe the recent progress of nanomaterials‐assisted metabolic analysis in IVD (Figure 1). The role of nanomaterials played in metabolic analysis is reviewed when coupled with different detection techniques, including electrochemical detection, optical detection, and mass spectrometric (MS) detection. We also highlight the nanomaterials‐assisted metabolic analysis toward IVD applications, from the perspectives of both the targeted biomarker quantitation and untargeted fingerprint extraction.

## 2 NANOMATERIALS‐ASSISTED METABOLIC ANALYSIS

Metabolites can be characterized by converting the corresponding abundance into electrochemical, optical, or mass‐to‐charge (*m*/*z*) ratio signals. Accordingly, the mainstream platform for metabolic analysis includes electrochemical detection, optical detection, and MS detection, each of which shows unique advantages and disadvantages (Table 1). Along with the rapid innovations of nanotechnology, various nanomaterials have been developed and fully engaged with detection platforms to ameliorate metabolic analysis.^[^ ^34^, ^35^, ^36^, ^37^ ^]^ In this section, we focus on the interaction of nanomaterials with specific detection techniques and emphasize the critical role nanomaterials play in metabolic analysis as summarized in Table 2.

**TABLE 1:** The comparison among three different detection platforms

**Detection platform**		**Advantages**	**Disadvantages**	**Future expectation**	**Ref**.
Electrochemical	Enzymatic electrode	Portability, easy operation, relatively high specificity, fast analytical speed	Low stability and reproducibility, low detection limits, low detection throughput, high cost	Improved sensitivity to detect analytes in bio‐specimens	^[^ ^38^, ^39^, ^40^, ^41^, ^42^, ^43^, ^44^, ^45^, ^46^, ^47^ ^]^
	Non‐enzymatic electrode	Portability, easy operation, good stability and reproducibility, cost‐efficiency, fast analytical speed, label‐free	Low selectivity, low detection limits, low detection throughput		
Optical	Fluorescence	High selectivity, high detection limits, good reproducibility	Not portable, complex operation, time‐consuming, low stability, low detection throughput	High detection throughput to characterize the metabolic fingerprints of bio‐specimens	^[^ ^64^, ^65^, ^66^, ^67^, ^68^, ^69^, ^70^, ^71^, ^72^, ^73^, ^74^, ^75^ ^]^
	Colorimetry	Portability, easy operation, cost‐efficiency, fast analytical speed	Low selectivity, low detection limits, low stability, and low reproducibility, low detection throughput		
	Raman spectrometry	Ultrahigh detection limits, fast analytical speed, good stability, high detection throughput, label‐free, cost‐effective	Not portable, complex operation, low reproducibility		
Mass spectrometric	GC/LC‐MS	Ultrahigh detection limits, good stability and reproducibility, high detection throughput, label‐free, accurate molecular identification	Not portable, complex operation, time‐consuming, high cost	Improved molecular identification for new biomarker discovery and minimized equipment size for POCT use	^[^ ^77^, ^78^, ^79^, ^80^, ^81^, ^82^, ^83^, ^84^, ^85^, ^86^, ^87^, ^88^, ^89^, ^90^, ^91^, ^92^, ^93^ ^]^
	LDI MS	Ultrahigh detection limits, fast analytical speed, good stability and reproducibility, high detection throughput, label‐free, cost‐effective, accurate molecular identification	Not portable, complex operation		

**TABLE 2:** Nanomaterials‐assisted metabolic analysis based on three different detection platforms

	**Nanomaterials**			**Metabolic analysis**			
**Detection platform**	**Function**	**Property**	**Type**	**Analytes**	**LOD [µmol]**	**Linear range [µmol]**	**Ref**.
Electrochemical	Enzyme immobilization	Large surface area, high electrical conductivity	Graphene frameworks	Glucose	—		—
			CNT array	Uric acid	1	1	^[^ ^49^ ^]^
	Nanozymes	High stability, simple synthesis, cost‐efficiency	Nafion/GO‐AuNP hybrids	H_2_O_2_	1.9 × 10^–6^	1.9 × 10^–6^	^[^ ^53^ ^]^
			Au@PtNP/GO		1.62	1.62	^[^ ^54^ ^]^
	Substrates of non‐enzymatic electrodes	Direct electrocatalytic oxidation, fast response speed, good reproducibility	NiCo‐LDH@Au/Cu	Glucose	0.23	0.23	^[^ ^58^ ^]^
			Cu*_x_* Co*_y_* O_4_ nanowire framework thin films	Glucose in serum	1.36	1.36	^[^ ^59^ ^]^
Optical	Probe for fluorescence spectrometry	FRET process, stable photoluminescence, biocompatibility	N‐GQDs	Cysteine	0.05	0.05	^[^ ^32^ ^]^
			Polymer dots	Phenylalanine	3.5	3.5	^[^ ^65^ ^]^
			Cs‐GQDs	Glucose, H_2_O_2_	0.025, 0.023	0.025, 0.023	^[^ ^69^ ^]^
			MoS_2_ QDs		2.0 × 10^–6^/4.3 × 10^–5^	2 × 10^–6^/4.3 × 10^–5^	^[^ ^70^ ^]^
	Nanozyme for colorimetry	Catalytic activities	His‐AuNCs	PPi, ATP, ADP	5,600/8,400/23,000	5,600/8,400/23,000	^[^ ^72^ ^]^
			Plasmonic Au NPs	Glycemic, uric acid, cholesterol	1,250/0.1/0.1	1,250/0.1/0.1	^[^ ^73^ ^]^
			Microarrayed MoS_2_	Lactate	4.55 × 10^3^ [µmol L^–1^]	4.55 × 10^3^ [µmol L^–1^]	^[^ ^74^ ^]^
	Substrates for Raman spectrometry	Plasmonic resonance effect	The superlattice of Au NPs	Extracellular metabolites	1	—	^[^ ^75^ ^]^
			Au/ZnO nanorods	Lactate	2,000	—	^[^ ^41^ ^]^
			Fe_3_O_4_@Ag NPs	ATP, lactate	1 × 10^–8^	—	^[^ ^50^ ^]^
MS	In‐situ pretreatment of metabolites	Porous network, high extraction capacity	PS/SiO_2_@PDA nanofibers	Monohydroxy metabolite	1.0 × 10^–5^ to 1.5 × 10^–4^ [µmol L^–1^]	1.4 × 10^–4^ to 1.7 × 10^–1^ [µmol L^–1^]	^[^ ^78^ ^]^
			ZrO_2_/PITC	Catecholamines	1.6 × 10^–4^ to 2.4 × 10^–4^ [µmol L^–1^]	4.6 × 10^–3^ to 1.1 [µmol L^–1^]	^[^ ^79^ ^]^
	Matrix for LDI MS	High surface area, electronic conductivity	g‐C_3_N_4_ nanosheets	1‐Nitropyrene	<1 × 10^–12^	—	^[^ ^88^ ^]^
			FG nanosheets	Uric acid in urine	—		
			GDs	Glucose, myristic acid	<1 × 10^–15^	—	^[^ ^83^ ^]^
		Surface plasmonic resonance effect, increased local EM enhancement	PdPtAu alloys	Metabolites in plasma	—		
			Au nanoshells	Amino acids	3 × 10^–6^ to 3 × 10^–5^	—	^[^ ^22^ ^]^

### 2.1 Electrochemical detection

Electrochemical detection offers high selectivity and fast analytical speed for metabolite recognition. It also presents adaptability into assays of POCT due to the intrinsic merits of portability, easy operation, and low cost.^[^ ^38^, ^39^ ^]^ Electrochemical sensor measures the targeted metabolite molecules through current, resistance, and voltage variations within the electrodes. As a critical component in an electrochemical system, electrodes produce electric signal outputs by interacting with the targeted analytes. Recently, nanomaterials have inched their way into the design and fabrication of electrodes to improve electrochemical responses, due to large surface area (e.g., various nanosheets^[^ ^27^, ^28^, ^29^, ^30^, ^31^, ^32^, ^33^ ^]^) and superior electrochemical properties (e.g., carbon nanotubes^[^ ^40^, ^41^, ^42^, ^43^ ^]^ and silicon nanowires^[^ ^44^, ^45^, ^46^, ^47^ ^]^). Typically, the nanomaterial‐based electrodes for metabolic analysis can be subclassified into three types according to the material functions, including (1) facilitating enzymatic reactions of the original electrodes; (2) serving as nanozymes with enhanced catalytic activities for replacing natural enzymes; and (3) acting as substrates of non‐enzymatic electrodes with direct electrocatalytic oxidation and good stability.

**FIGURE 2:**
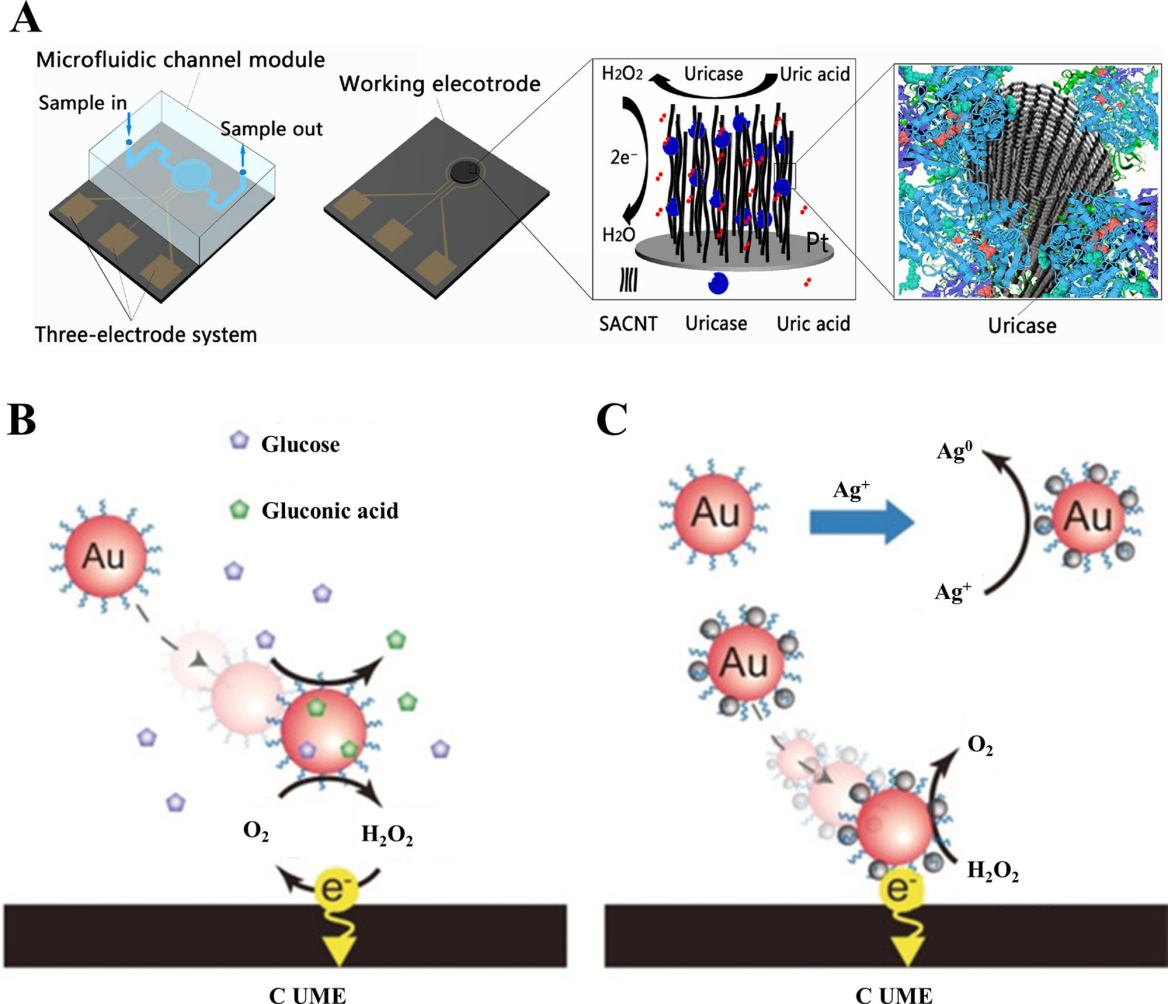
(A) Schematic illustration of the design and construction of the 3D super‐aligned carbon nanotube array for the electrochemical sensing of uric acid. Reproduced with permission.^[^ ^49^ ^]^ Copyright 2021, Elsevier. The schemes in (B) and (C) illustrate the electrochemical detection of glucose via the current variation. Here, the collision of a single gold nanoparticle at a carbon ultramicroelectrode surface was resulted from the catalytic reaction and contributed to the current variation for glucose sensing. Reproduced with permission.^[^ ^52^ ^]^ Copyright 2018, Wiley‐VCH

Nanomaterials can facilitate catalysis‐based electrochemical sensing via immobilizing enzymes.^[^ ^48^ ^]^ Providing specific recognition and catalysis toward metabolites, enzymes have been exerted as the building blocks in electronical sensing systems. However, the enzymatic electrodes face challenges of unsatisfied loading amount and low stability of enzymes. To tackle the above obstacles, nanomaterials with precisely‐designed nanostructures can improve the enzyme immobilization rate and the related electron transfer process.^[^ ^48^ ^]^ For instance, Yang et al. prepared an electrode by immobilizing uricase with a 3D super‐aligned carbon nanotube array, offering a high enzyme density of uricase and large contact area with reactants of uric acid (Figure 2A).^[^ ^49^ ^]^ As a result, this enzymatic electrode demonstrated high detection sensitivity toward uric acid, with a limit of detection (LOD) of 1 µmol within a wide linear range of 100–1000 µmol.

Other than enhancing catalytic activity by increasing the loading rate of natural enzymes, nanomaterials consisting of transition metals (e.g., Fe and Cu)^[^ ^50^, ^51^ ^]^ and noble metals (e.g., Au and Pt)^[^ ^52^, ^53^, ^54^ ^]^ possess an intrinsic enzyme‐mimicking ability, thus can be employed as nanozymes to improve the detection performance of electrochemical sensors. As an alternative to natural enzymes, nanozyme‐based electrodes display superiorities of high stability, simple synthesis, and cost efficiency in metabolic analysis. Specifically, noble metal nanoparticles (NPs), like Au NPs, present both oxidase and peroxidase activities for electrochemical detection of glucose (Figure 2B,C).^[^ ^52^ ^]^ The common strategy for incorporating nanozymes into electrochemical sensing systems is to develop nanomaterial hybrids with synergistic effect to enhance detection sensitivity.^[^ ^53^, ^54^, ^55^, ^56^ ^]^ For instance, the nafion/graphene oxide‐gold nanoparticle (GO‐AuNP) hybrid developed by Jin et al. served as peroxidase mimics for building the electrode and detecting H_2_O_2_, which displayed an estimated LOD of 1.9 nmol within a linear detection range of 10–10 mmol.^[^ ^53^ ^]^ In addition to monometallic nanozymes, many attempts were devoted to investigating the multi‐metallic nanomaterials for electrochemical sensing. Ko et al. fabricated Au@PtNP/GO as peroxidase mimics for H_2_O_2_ electrochemical detection with a linear range of 1–3000 µmol and LOD of 1.62 µmol.^[^ ^54^ ^]^ However, compared to monometallic nanozymes, a performance loss was witnessed by using Au@PtNP/GO, owing to the lack of systematic optimization of multi‐metallic electrodes. Therefore, more efforts are required to explore the synergic effect among different metal elements toward a satisfied electrochemical biosensing of metabolites.

**FIGURE 3:**
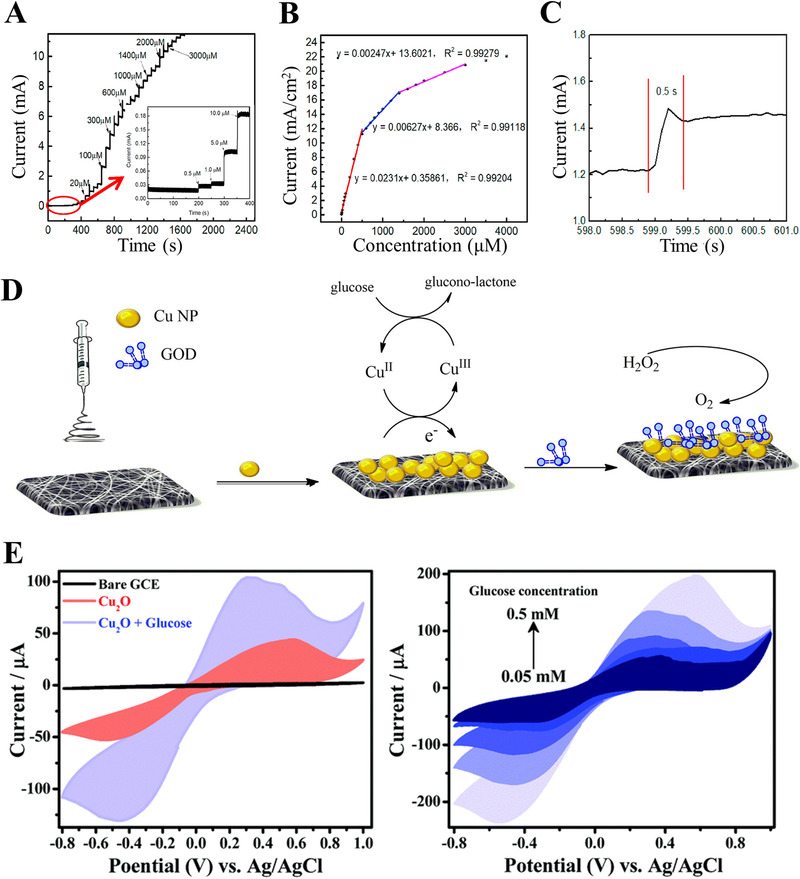
(A–C) An electrochemical non‐enzymatic glucose sensor constructed by NiCo‐LDH nanoflake arrays‐supported Au nanoparticles (NPs) on copper foam (NiCo‐LDH@ Au/Cu). Notably, the nanomaterial‐based electrode exhibits sensitive detection of glucose with a low detection limit of 0.23 µmol, due to the synergistic effect of three‐dimensional architecture of Cu foam, high electrocatalytic activity of Au NPs, and NiCo‐LDH nanoflake arrays. Reproduced with permission.^[^ ^58^ ^]^ Copyright 2021, Elsevier. (D) Construction and comparison of a non‐enzymatic electrode and an enzymatic electrode, based on Cu nanoparticle‐coated polyacrylonitrile electrospun nanofibrous. Reproduced with permission.^[^ ^51^ ^]^ Copyright 2021, Elsevier. (E) Cyclic voltammograms of as‐synthesized Cu_2_O octahedron modified glassy carbon electrode for glucose detection. Reproduced with permission.^[^ ^57^ ^]^ Copyright 2021, Royal Society of Chemistry

To push this field forward, non‐enzymatic electrodes have emerged as an important research direction in electrochemical detection by addressing the limitations of enzymatic electrodes.^[^ ^57^ ^]^ In particular, nanomaterials can strengthen the non‐enzymatic electrochemical detection with direct electrocatalytic oxidation, fast response speed, good reproducibility. For example, Shen et al. applied 3D architecture of Cu foam for introducing the noble metal by galvanic replacement and then modified with NiCo‐layered double hydroxide materials, making full use of their electrocatalytic performance and electronic/ionic transport for electrode construction. The as‐prepared electrochemical biosensor achieved accurate glucose detection, affording a relatively lower LOD of 0.23 µmol and a broader linear range of 0.5–3000 µmol compared to the enzymatic based electrode (Figure 3A–C).^[^ ^58^ ^]^ To quantitively evaluate the detection performance of non‐enzymatic and enzymatic electrodes, Apetrei et al. fabricated a non‐enzymatic electrode by coating Cu NPs onto a polyacrylonitrile (PAN) electrospun nanofibrous (NFs) assembly for glucose detection and observed a LOD of 5.9 µmol within a linear range of 20–1000 µmol.^[^ ^51^ ^]^ In contrast, the authors further modified the PAN NFs/Cu NPs by the cross‐linking of the glucose oxidase to obtain an enzymatic electrode (Figure 3D). As a result, the non‐enzymatic glucose electrode afforded three times of enhancement in detection sensitivity as compared to the enzymatic electrode, due to amplification provided by nanofibrous PAN matrix and Nafion coating. Except for sensitivity, detection selectivity also plays an important role in analytical science. It is noteworthy that the selectivity of non‐enzymatic electrodes results from the design and optimization of material components, rather than the inherent catalytic selectivity of enzymatic electrodes.^[^ ^59^, ^60^, ^61^ ^]^ For instance, the well‐established Cu*_x_* Co*_y_* O_4_ nanowire framework thin‐films by Xu et al. enhanced the detection selectivity of glucose in serum based on abundant electro‐active sites and channels for ions transfer.^[^ ^59^ ^]^ Regarding the substrate choice, the transition metal‐based non‐enzymatic electrode (e.g., Cu^[^ ^57^ ^]^) offered preferable electrocatalytic ability and low cost and served as a better candidate (Figure 3E) than the previous noble metal substrate for building the novel electrochemical biosensor.

In general, nanomaterials‐assisted electrochemical sensing systems were constructed with enhanced sensitivity and selectivity for metabolic analysis. Further considering the applicability for POCT, nanomaterials‐assisted electrochemical sensor provides an opportunity to be extended to real‐case applications.

### 2.2 Optical detection

Optical detection allows highly sensitive, cost‐effective, and simple metabolite measurements, promising clinical diagnostics under various circumstances.^[^ ^62^, ^63^ ^]^ Further enhanced by designed nanomaterials, the optical biosensors can quantify metabolites in biospecimens at a wide dynamic range and low detection limit. The key roles nanomaterials acting in optical detection can be defined as probes with unique fluorescence resonance energy transfer (FRET) process for fluorescence spectrometry, as nanozyme with catalytic activities for visible spectrometry, and as substrates with plasmonic resonance effect for Raman spectrometry.

**FIGURE 4:**
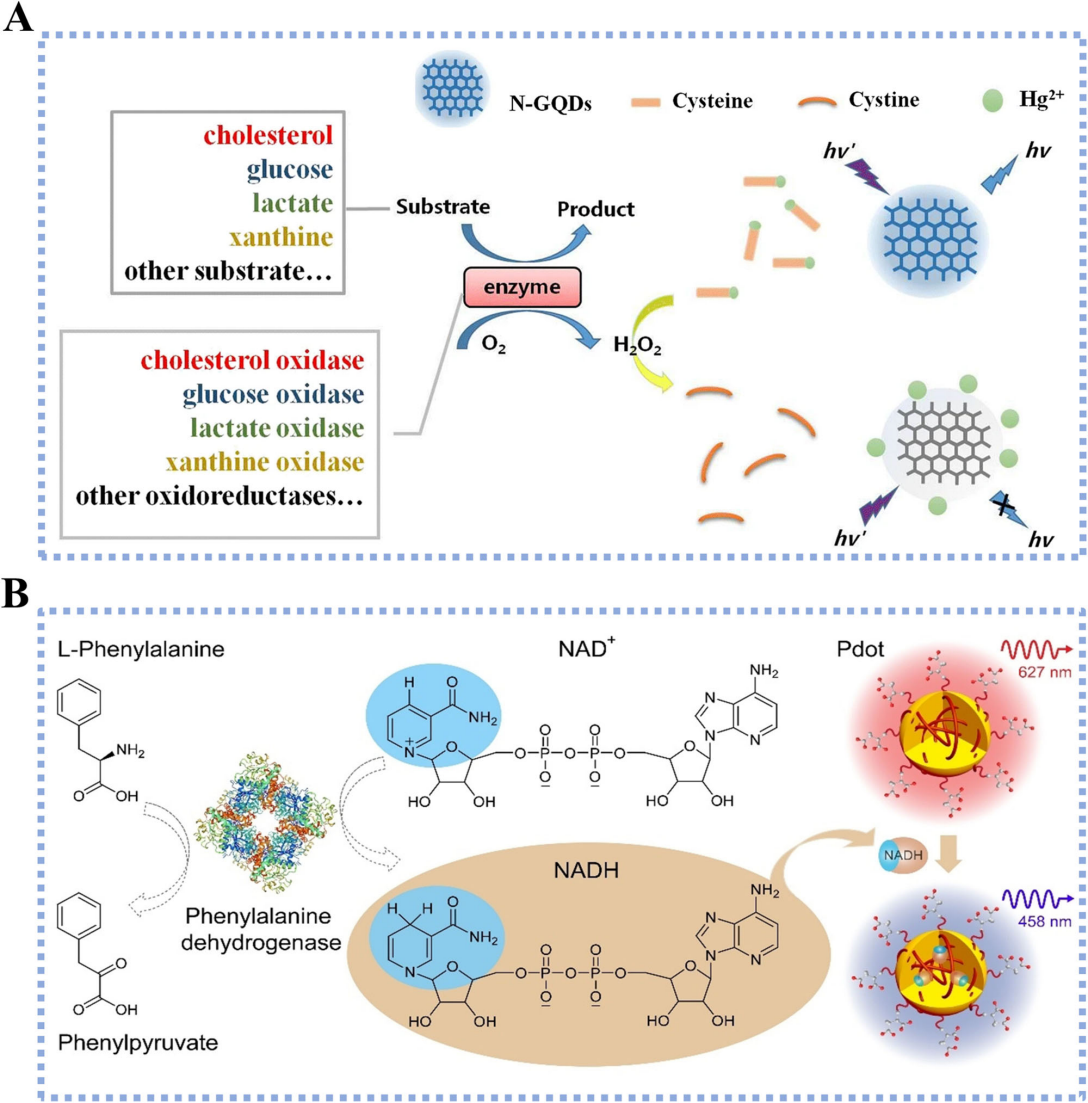
(A) Schematic illustration of a fluorescence‐based sensing system by using nitrogen‐doped graphene quantum dot as the fluorescence probe. The platform was designed to detect various metabolites, including cholesterol, glucose, lactate, xanthine, and other small molecules, Reproduced with permission.^[^ ^64^ ^]^ Copyright 2020, Springer Nature. (B) A NAD(P)H‐sensitive polymer dot (Pdot) biosensor for point‐of‐care monitoring of phenylalanine. Here, the Pdot was combined with a phenylalanine‐specific NAD(P)H‐dependent enzyme to generate NAD(P)H. In turn, the NAD(P)H quenched the fluorescence emission of Pdot at 627 nm and fluoresced at 458 nm upon ultraviolet illumination. Reproduced with permission.^[^ ^65^ ^]^ Copyright 2021, Wiley‐VCH

For fluorescence detection, metabolic signals are measured by spectrum variation, with nanomaterials serving as a contributor in fluorescence quenching and enhancement.^[^ ^64^, ^65^ ^]^ In particular, QDs^[^ ^66^, ^67^, ^68^, ^69^, ^70^ ^]^ as zero‐dimensional (0D) nanomaterials have been explored as the fluorescence probes due to the unique FRET process, stable photoluminescence, and biocompatibility.^[^ ^71^ ^]^ A typical fluorescence‐based metabolite sensing process with the introduction of nanomaterials is illustrated in Figure 4A.^[^ ^64^ ^]^ The nitrogen‐doped graphene QDs (N‐GQDs) were combined with V_2_O_5_ nanosheets for cysteine detection with LOD reaching 50 nmol, where the V_2_O_5_ nanosheets functioned as the fluorescence quencher and cysteine recognizer.^[^ ^32^ ^]^ Specifically, the adsorption of Hg^2+^ on the N‐GQDs surface resulted in fluorescence quenching due to the effective electron transfer, and the presence of cysteine recovered the fluorescence by blocking the transfer process. As the existence of metabolite further led to the fluorescence quenching through the related enzymatic oxidation, the metabolite detection thus could be achieved by observing the change of fluorescence. In addition, fluorescence probes can also be designed with FRET effect for detecting and quantifying the metabolites. Chen et al. combined the NAD(P)H‐sensitive polymer dots (Pdots) with the metabolite‐specific NAD(P)H‐dependent enzyme (Figure 4B).^[^ ^65^ ^]^ Under ultraviolet (UV) illumination, the NAD(P)H generated from the metabolite oxidation led to the fluorescence quenching emitted at 627 nm and fluoresced at 458 nm by Pdots. The ratio of blue‐to‐red channel emission intensities was further applied for detecting and quantifying the phenylalanine with LOD of 3.5 µmol. As doping heteroatoms can tune the optical property of nanomaterials, Wen et al. fabricated cesium‐doped GQDs (Cs‐GQDs), resulting in a clear blue shift of the fluorescence emission peak.^[^ ^69^ ^]^ They also explored the FRET between 2,3‐diaminophenazine (a yellow fluorescent) and the Cs‐GQDs and achieved the sensitive detection of glucose (LOD of 25 nmol) and H_2_O_2_ (LOD of 23 nmol). To further improve the detection sensitivity of FRET‐based detection systems, previous efforts turned to surface functionalization of QDs using organic groups. As an example, Mahle et al. used Pseudomonas aeruginosa for green synthesis and organic functionalization of MoS_2_ QDs, which achieved the ultrasensitive glucose detection down to pico‐molar level with controlled chemical/optical characteristics.^[^ ^70^ ^]^

Colorimetry features visual detection of metabolites via color variations or UV absorption spectrum without complicated equipment.^[^ ^72^, ^73^, ^74^ ^]^ The functionalized noble metals like Au offer the intrinsic peroxidase‐like activity, which promotes superoxide anion formation and electron transfer and can be exploited for colorimetric assays. For example, the phosphate‐containing metabolites can be detected by blocking the superoxide anion production and related electron transfer process. Based on the above mechanism, Chen et al. developed a colorimetric on‐off switch for detecting inorganic pyrophosphate ion (PPi), adenosine triphosphate (ATP), and adenosine diphosphate (ADP), with LOD of 5.6/8.4/23 nmol and a linear range from 0.01–10 µmol/0.01–18 µmol/0.05–60 µmol, respectively.^[^ ^72^ ^]^ Notably, there were some exciting attempts that combined microfluidic systems with colorimetric assays to enable simultaneous metabolite detection.^[^ ^73^, ^74^ ^]^ Park et al. fabricated an optoelectronic biosensor chip by integrating the microarrayed MoS_2_ with microfluidic chambers for enzymatic reaction, allowing the colorimetric detection of lactate with LOD of 0.51 ng mL^–1^.^[^ ^74^ ^]^ In addition, Phinheiro et al. developed a microfluidic paper‐based analytical device relying only on the plasmonic transduction of Au NPs and further improved the accessibility and cost‐efficiency of colorimetric assays.^[^ ^73^ ^]^ This system was capable of the multiparametric quantification of glycemic (LOD of 1.25 mmol), uric acid (LOD of 0.1^ ^µmol), and cholesterol (LOD of 0.1 µmol). While the advanced colorimetric system has fulfilled the current needs in measuring specific metabolites (e.g., glucose for diabetes), designed nanomaterials are still required for an optimized LOD for metabolite to achieve broader applications toward IVD.

**FIGURE 5:**
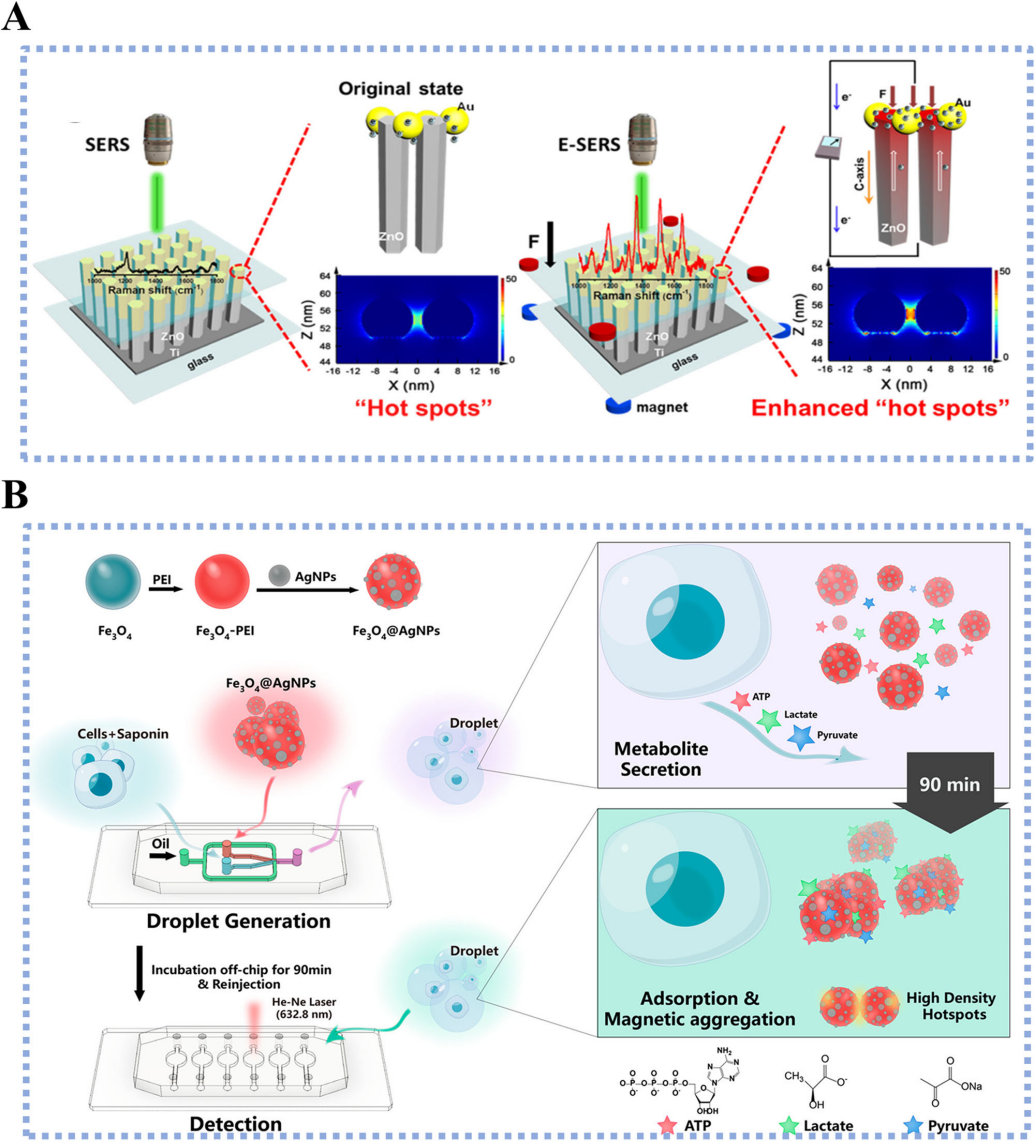
(A) Illustration of the fabrication of one‐dimensional asymmetric Au/ZnO nanorods for Raman detection of metabolites. Reproduced with permission.^[^ ^41^ ^]^ Copyright 2021, American Chemical Society. (B) Workflow of surface enhanced Ramen scattering‐microfluidic droplet platform for encapsulating single cells and detecting metabolites (e.g., ATP, lactate, and pyruvate) at single‐cellular resolution. Particularly, magnetic force was introduced into this system by using Fe_3_O_4_ particles, to manipulate and sort single cells. Reproduced with permission.^[^ ^50^ ^]^ Copyright 2019, American Chemical Society

Raman spectroscopy provides a structural fingerprint of metabolites by determining their vibrational modes, while low detection sensitivity of Raman spectroscopy places a challenge in biomedical applications. In particular, surface enhanced Raman spectrometry (SERS) increases Raman signal intensity with localized electromagnetic (EM) field enhancement on plasmonic nanostructures. For example, Plou et al. reported a nanostructured plasmonic substrate composed of the superlattice of Au NPs as SERS substrate, which served as the enhancement contributor for metabolic signal and achieved the detection of extracellular metabolites (e.g., kynurenine, tryptophan, and purine derivatives).^[^ ^75^ ^]^ However, the practical use of noble metal materials has been restricted by their intrinsic high cost and unsatisfied mechanical strength. Recently, researchers intended to combine the noble metals with transition metal oxides to improve the cost‐efficiency and mechanical strength. Xu et al. developed an electrically modulated substrate for SERS by integrating ZnO nanorods with asymmetric Au NPs. The substrate offered a SERS signal enhancement of 6.7 times with the efficacious separation of electron‐hole pairs, owing to the existence of Schottky barrier at the material interfaces (Figure 5A).^[^ ^41^ ^]^ They further integrated this substrate into a wearable flexible sensor, affording a sensitive and real‐time monitoring of lactate with LOD of 2 mmol. Similarly, Sun et al. decorated Fe_3_O_4_ microspheres with Ag NPs to obtain a metal‐magnetic composite substrate (Fe_3_O_4_@Ag NPs) for metabolite detection at single‐cell resolution (Figure 5B). Specifically, Fe_3_O_4_ microspheres with magnetic characteristics assisted the cell separation and avoided the endocytosis effect during metabolic analysis.^[^ ^50^ ^]^ The Fe_3_O_4_@Ag NPs supported SERS detection achieved the detection of ATP with LOD of 0.1 pmol, lactate with LOD of 0.01 pmol, and pyruvate with LOD of 0.01 pmol, displaying a several order of magnitude improvement compared to the electrochemical and other optical assays. While ultrasensitive, the current nanomaterial‐assisted SERS detection is limited to the targeted detection of specific metabolites. It is expected that the untargeted metabolic fingerprinting of bio‐specimen will be a breakthrough point in SERS detection, after addressing the existed challenges in molecule identification via decoding molecular vibrations.

### 2.3 MS detection

MS is recognized as the primary tool for metabolic analysis by demonstrating the superiority in sensitivity, accuracy, detection throughput, and sample usage.^[^ ^76^ ^]^ Specifically, MS provides a comprehensive characterization for metabolites by acquiring the specific *m*/*z* ratio with unique ion fragmentation pattern.^[^ ^14^ ^]^ In the recent 5 years, nanomaterials were introduced into MS system to assist metabolic analysis with enhanced selectivity and sensitivity.

**FIGURE 6:**
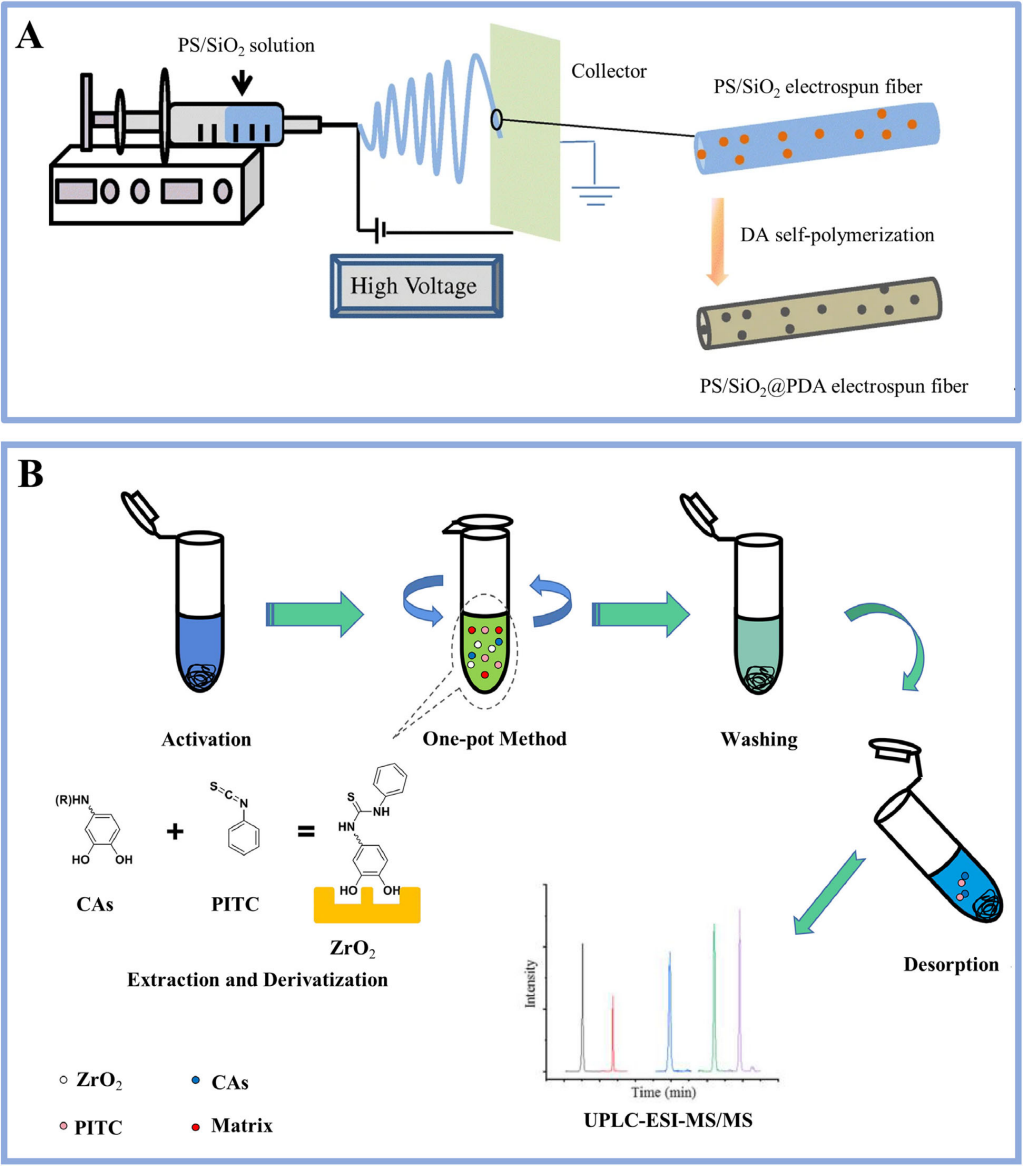
(A) Preparation of polydopamine‐modified polystyrene/silica electrospun nanofibers as an adsorbent for monohydroxy metabolites of polycyclic aromatic hydrocarbons prior to MS analysis. Reproduced with permission.^[^ ^78^ ^]^ Copyright 2019, Springer Nature. (B) Experimental protocol of simultaneous extraction/derivatization strategy for effective pretreatment of catecholamines for MS use. In particular, commercial zirconium oxide NPs were employed for the selective capturing of cis‐diol containing catecholamines to remove the biological interferences and to improve the chromatographic separation. Reproduced with permission.^[^ ^79^ ^]^ Copyright 2021, Wiley‐VCH

To enhance detection selectivity, nanomaterials with designed surface structures and chemical modifications shed light on the in‐situ pretreatment of metabolites from different types of biospecimens before MS analysis.^[^ ^77^ ^]^ Typically, nanomaterials enrich targeted metabolites and remove interference biomolecules (e.g., proteins and nucleic acids) to enhance the detection selectivity of metabolic analysis, which is critical to characterizing real‐case bio‐samples in clinical settings. The nanomaterials with porous network and extraction capacity can be employed as an adsorbent for enriching low‐abundance metabolites. For instance, Chen et al. reported polydopamine modified polystyrene/silica electrospun nanofibers for targeted enrichment of monohydroxy metabolites (Figure 6A).^[^ ^78^ ^]^ These nanofibers with high porosity and hydrophilicity contributed to the excellent enrichment factors of 24 and 112 and LOD of 2 and 21 pg mL^–1^ for 3‐hydroxyphenanthrene and 1‐hydroxypyrene, respectively. Except for purification and preconcentration, the derivatization process is also important prior to MS measurements to improve ionization efficiency and chromatographic separation.^[^ ^79^, ^80^ ^]^ In this case, nanomaterials were designed to be coupled with gas/liquid chromatography to simplify the pretreatment procedures for metabolic analysis. Very recently, Chen et al. developed ZrO_2_ and phenyl isothiocyanate hybrids for the pretreatment of catecholamines (CAs).^[^ ^79^ ^]^ The hybrids could capture CAs selectively by removing biological interferences and realized the derivatization of CAs (Figure 6B). Such a simultaneous extraction/derivatization method based on nanomaterials guaranteed an enhanced sensitivity in the detection of urinary CAs with LOD of 0.035–0.050 ng mL^–1^.

**FIGURE 7:**
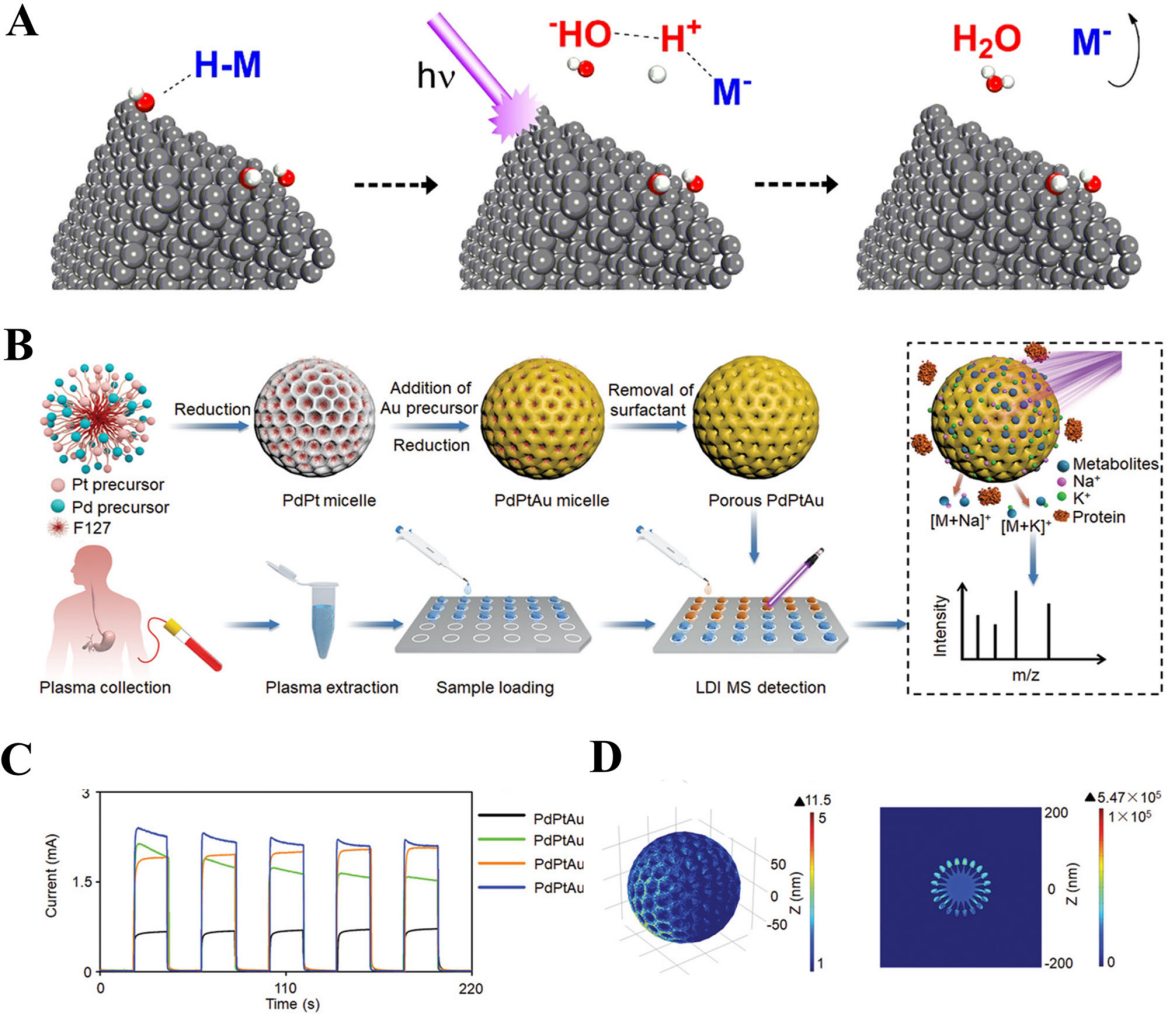
(A) Hydroxyl‐group‐dominated graphite dots as matrices to enhance the metabolic analysis by laser desorption/ionization (LDI) MS. Reproduced with permission.^[^ ^83^ ^]^ Copyright 2017, American Chemical Society. (B) Overall schematics for detecting metabolites from complex plasma samples using PdPtAu alloys. (C) Photocurrent response of PdPtAu alloys and (D) its finite‐different time‐domain simulation of localized electromagnetic distribution. Reproduced with permission.^[^ ^89^ ^]^ Copyright 2021, Wiley‐VCH

To enhance detection sensitivity, nanomaterials serving as matrices benefit desorption/ionization process, thus were widely adopted in matrix‐assisted laser desorption/ionization MS (MALDI MS).^[^ ^81^, ^82^, ^83^, ^84^ ^]^ In general, nanomaterials can influence the MS sensitivity from the following two aspects: (1) high electronic conductivity facilitating molecular ionization process; and (2) surface plasmon resonance improving laser energy transfer. Specifically, carbon‐based nanomaterials with high surface area and electronic conductivity were developed as one of the most popular MS matrices.^[^ ^85^, ^86^, ^87^ ^]^ However, the materials have long been concerned about the thermal instability and self‐dissociations under laser ablation, which leads to interference signals shown in mass spectrum and contamination to ion source. Lin et al. developed ultrathin graphitic carbon nitride (g‐C_3_N_4_) nanosheets as matrix for MS use.^[^ ^88^ ^]^ The authors observe few interferent background noise from C_3_N_4_ nanosheets and witnessed clear spectrum from the targeted metabolites. The C_3_N_4_ nanosheets exhibited high LDI efficiency due to the preferable electronic properties of nitrogen, thus realizing sensitive detection of 1‐nitropyrene with LOD down to 1 pmol. Sun et al. also addressed the contamination of ion sources by using fluorographene (FG) nanosheets as matrix.^[^ ^29^ ^]^ FG nanosheets derived from graphene possess the continuous π‐conjugated network for assisting an efficient laser absorption and energy transfer, thus achieving uric acid detection from urine sample (concentration of 4 mM). In addition to two‐dimensional carbon‐based matrices, the 0D graphite dots (GDs) could also serve as the MS matrix for metabolite detection, with high aqueous solubility, good biocompatibility, and salt tolerance. For instance, Shi et al. illustrated the MS detection process by applying hydroxyl‐group‐dominated GDs as the inorganic matrix, as shown in Figure 7A.^[^ ^83^ ^]^ The hydroxyl‐group‐dominated GDs exhibited strong UV absorption and photochemical properties and contributed to the real‐time monitoring of metabolites (e.g., glucose and myristic acid) with ultrahigh sensitivity (LOD < 1 fmol).

Unlike carbon‐based nanomaterials, metal‐based nanomaterials have been extensively explored as the MS matrices mainly due to the surface plasmonic resonance effect.^[^ ^89^, ^90^, ^91^, ^92^, ^93^ ^]^ Both the compositions and nanostructures of metal‐based nanomaterials decide the LDI efficiency for MS analysis. As for the composition, nanocomposites have attracted much attention for exploring the synergistic effects of different metallic components. Su et al. developed a trimetallic MS matrix of mesoporous PdPtAu alloys with elemental composition optimized (Figure 7B–D).^[^ ^89^ ^]^ They verified the potentials of trimetallic materials as MS matrix with an enhanced localized EM field under laser excitation, showing a maximum 8.12 times of increase compared to monometallic matrices. As a result, the PdPtAu alloy‐assisted MS achieved direct molecular fingerprinting of low‐abundance metabolites, by consuming 500 nl of native plasma samples only. For the structure, plasmonic nanoshells were considered the better alternatives to materials of other morphologies, with increased local EM enhancement. In detail, Wei et al. adopted the finite‐different time‐domain simulations to investigate the influence by diverse structures for metabolite detection.^[^ ^22^ ^]^ They first synthesized Au‐based nanomaterials with different morphologies, such as core‐shells and solid particles. The simulation results illustrated that Au nanoshells possessed unique antibonding modes for hot carrier generation under laser irradiation. Consistently, the Au nanoshells displayed the desirable sensitivity in metabolite detection with LOD of 3–30 pmol for amino acids (e.g., valine and methionine).

Among the numerous developed materials being used in MS, anisotropic nanomaterials possessing unique physicochemical properties are worthy of further exploration, potentially offering insights into metabolic analysis by MS techniques.

## 3 THE APPLICATION OF METABOLIC ANALYSIS IN IVD

Metabolic analysis of diverse bio‐specimens has garnered significant interest as a promising tool in IVD applications. In this section, we introduced the targeted quantitation of metabolite biomarkers, followed by the untargeted fingerprinting of metabolic signature (Table 3).

**TABLE 3:** The applications of metabolic analysis in IVD

**Type of metabolic analysis**		**Detected biospecimens**	**Related disease**	**Nanomaterials**	**Analytical tools**	**Diagnostic performance**	**Ref**.
Targeted quantitation of metabolite biomarkers	Glucose	Blood	Hyperglycemia, diabetes	NiCo‐LDH@Au/Cu, Cu*_x_* Co*_y_* O_4_ nanowire framework thin films, Cs‐GQDs, MoS_2_ QDs	Non‐enzymatic electrode, colorimetry	—	^[^ ^58^, ^59^, ^69^, ^70^ ^]^
		CSF	Postoperative brain infection	Ag nanoshell, 2D‐Au NPs array, MOSF@GNPs@oxidases nanoreactor	LDI MS, SERS	Sen/Spe/AUC of 0.816/0.882/0.961	^[^ ^97^, ^98^, ^99^ ^]^
	Uric acid	Serum	Gout	SACNT array	Electrochemical biosensor	—	^[^ ^49^ ^]^
		Urine	Hyperuricemia	Alg@QDs‐UOx MSs	Optical sensor	Acc of 100%	^[^ ^100^ ^]^
Untargeted fingerprinting of metabolic signatures		Plasma	Diabetic retinopathy	V_2_O_5_ nanorods	LDI MS	Sen/Spe/AUC of 0.90/0.83/0.926 in validation cohort	^[^ ^109^ ^]^
		Serum	Gynecological cancer	FeOOH@ZIF‐8	LDI MS	AUC of 0.998–0.999	^[^ ^111^ ^]^
		Urine	Kidney disease	Polymer@Ag	LDI MS	AUC of 0.86	^[^ ^116^ ^]^
		Cell	Malignant melanoma	Multibranched Au NPs	SERS	Acc of 100%	^[^ ^118^ ^]^
		Exosome	Lung cancer	Plasmonic gold chip	LDI MS	*R* ^2^ = 0.994, *Q* ^2^ = 0.716, *p* < 0.0001 (by OPLS‐DA)	^[^ ^121^ ^]^

### 3.1 Targeted quantitation of metabolite biomarkers

Metabolite biomarkers in biospecimens are of fundamental importance in diagnostics by presenting downstream feedbacks resulting from genomics and proteomics. Numerous efforts have been applied to integrating nanomaterials with analytical chemical approaches for the accurate quantitation of targeted metabolite biomarkers.^[^ ^94^, ^95^, ^96^, ^97^, ^98^, ^99^, ^100^, ^101^, ^102^, ^103^, ^104^, ^105^ ^]^

**FIGURE 8:**
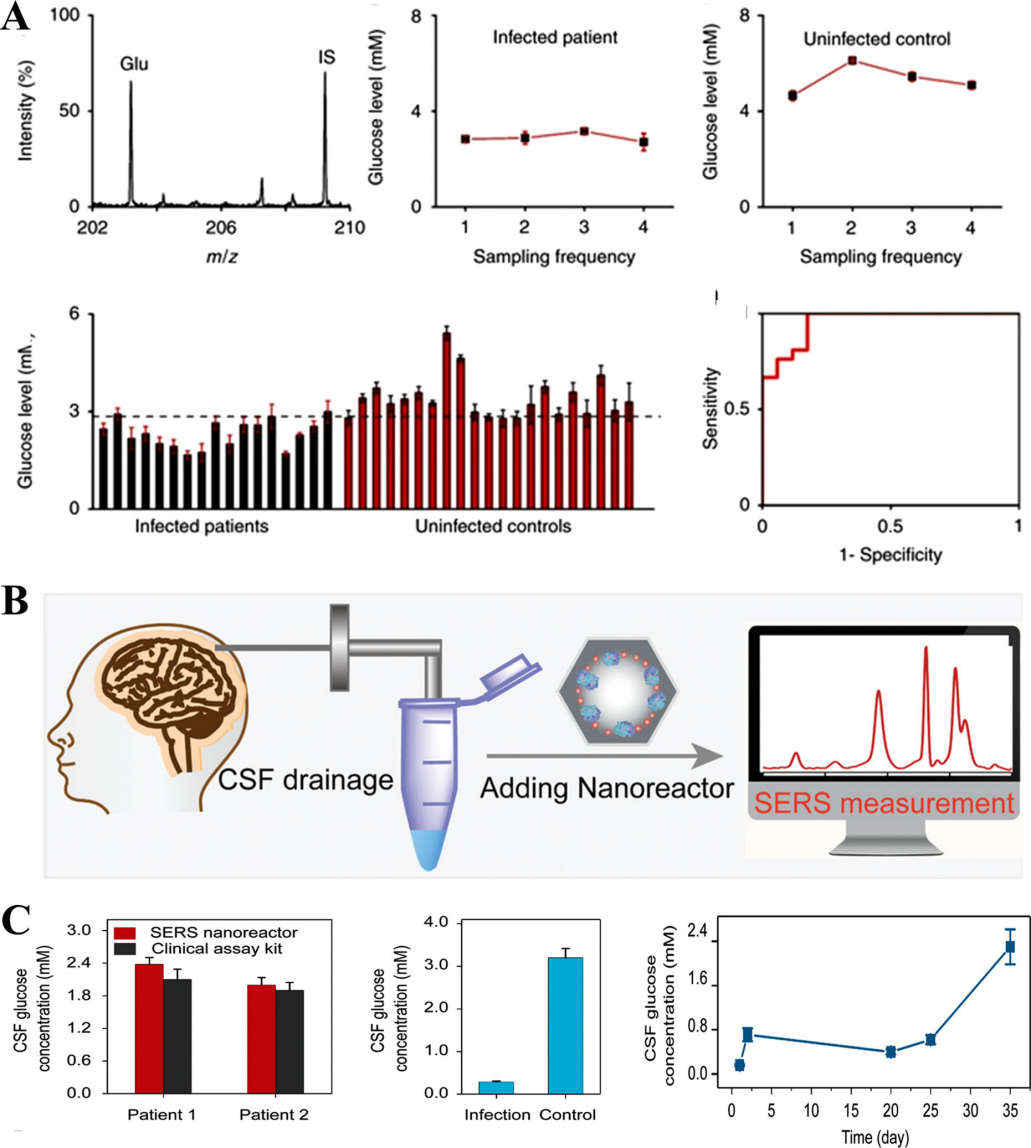
(A) Postoperative brain infection diagnosis based on glucose quantitation. The accurate quantitation of glucose was achieved by a plasmonic silver nanoshell‐assisted LDI MS. Reproduced with permission.^[^ ^97^ ^]^ Copyright 2017, Springer Nature. (B) Scheme of monitoring glucose in patient CSF using SERS with the aid of plasmonic nanoreactor. (C) Glucose concentrations in patient CSF were determined by both nanoreactor‐enhanced SERS and clinical test kit. Reproduced with permission.^[^ ^99^ ^]^ Copyright 2020, Wiley‐VCH

Among diverse metabolite biomarkers, glucose highly correlates with the diagnosis and management of diabetes.^[^ ^94^, ^95^, ^96^ ^]^ Its blood level above 7 mM can be considered as hyperglycemia and is indicative of diabetes, which can be accessible through electrochemical (e.g., non‐enzymatic electrode^[^ ^58^, ^59^ ^]^) and optical assays (e.g., colorimetry^[^ ^69^, ^70^ ^]^). However, glucose detection in cerebrospinal fluid (CSF) requires high sensitivity to support the identification of patients with postoperative brain infection. Accordingly, Huang et al. designed a plasmonic Ag nanoshell‐assisted LDI MS to measure the glucose levels in CSF samples from 17 infected patients and 21 uninfected patients.^[^ ^97^ ^]^ The optimized plasmonic silver nanoshells as matrix allowed LDI MS detection with high sensitivity and selectivity. After further introduction of stable isotopes of the targeted biomarker, the quantitation by Ag nanoshell‐assisted LDI MS was accurate with recovery rate of ∼107% and coefficients of variation (CV) of <17%, comparable with traditional biochemical approach with linear fit of 0.92. By consuming 500 nl of native CSF, they further achieved the precise diagnosis of postoperative brain infection, showing an optimized diagnostic sensitivity/specificity of 81.6/88.2% with the area‐under‐the‐curve (AUC) of 0.961 (Figure 8A). After that, Wang et al. developed self‐assembled Au nanoparticles arrays to address the oxidation issue of Ag nanoshells in LDI MS detection, which also showed the capability of glucose quantitation of CSF samples for detection and monitoring of brain infection.^[^ ^98^ ^]^ Furthermore, the glucose level in CSF was also quantitatively characterized by Liu et al. using a novel bio‐nanoreactor‐assisted SERS.^[^ ^99^ ^]^ The above bio‐nanoreactor maintained catalytic activities even under harsh conditions, which could also serve as a smart metabolite sensing platform by conversing analytes into H_2_O_2_. By using an internal spectral calibration, the radiometric platform consumed 20 µl of CSF from two patients and achieved accurate glucose quantitation with detected results of 2.3 and 2.1 mmol, agreeing with the results from clinical assay kit (*p* > 0.05). The authors also monitored the variation dynamics of CSF glucose as the patient received continuous antibiotic treatment for 35 days and discovered an increasing trend of glucose changing from 0.1 to 2.38 mmol (Figure 8B,C).

Uric acid is another essential biomarker as the end‐product of purine metabolism, the elevated serum level of which serves as an indicator for gout.^[^ ^73^ ^]^ Yang et al. measured the concentration of uric acid in the spiked serum sample of healthy volunteers using an electrochemical biosensor.^[^ ^49^ ^]^ This biosensor was fabricated by a super‐aligned carbon nanotube array, thus offering a high enzymatic density and larger contact area with analytes. As a real‐case demonstration, it achieved accurate quantitation of uric acid and showed a high consistency with a Food and Drug Administration‐approved electrochemical analyzer (*p* > 0.05 from paired t‐test). Moreover, the determination of uric acid in urine samples is also critical to reveal the human state. Lu et al. developed a sensor based on alginate hydrogel microspheres for detecting uric acid in urine samples from healthy controls and patients. They used a camera equipped in smartphone to differentiate patients from healthy volunteers based on the level of uric acid. The whole process could be completed within 10 min, achieving a classification accuracy of 100%.^[^ ^100^ ^]^ This device showed an improved adaptability toward POCT use.

Similar attempts were also reported for the detection of other metabolite biomarkers, including amino acids (e.g., cysteine),^[^ ^106^, ^107^ ^]^ lipids (e.g., cholesterol),^[^ ^101^, ^102^, ^103^ ^]^ vitamins (e.g., ascorbic acid),^[^ ^104^, ^105^ ^]^ and so forth. The introduction of nanomaterials aims at optimizing LOD for biomarker quantitation to satisfy the unmet diagnostic demand.

### 3.2 Untargeted fingerprinting of metabolic signature

Untargeted metabolic fingerprints provide a global and in‐time characterization of the human body toward IVD applications.^[^ ^108^ ^]^ Nanomaterials have been extensively used for metabolic fingerprinting from diverse bio‐specimens.

**FIGURE 9:**
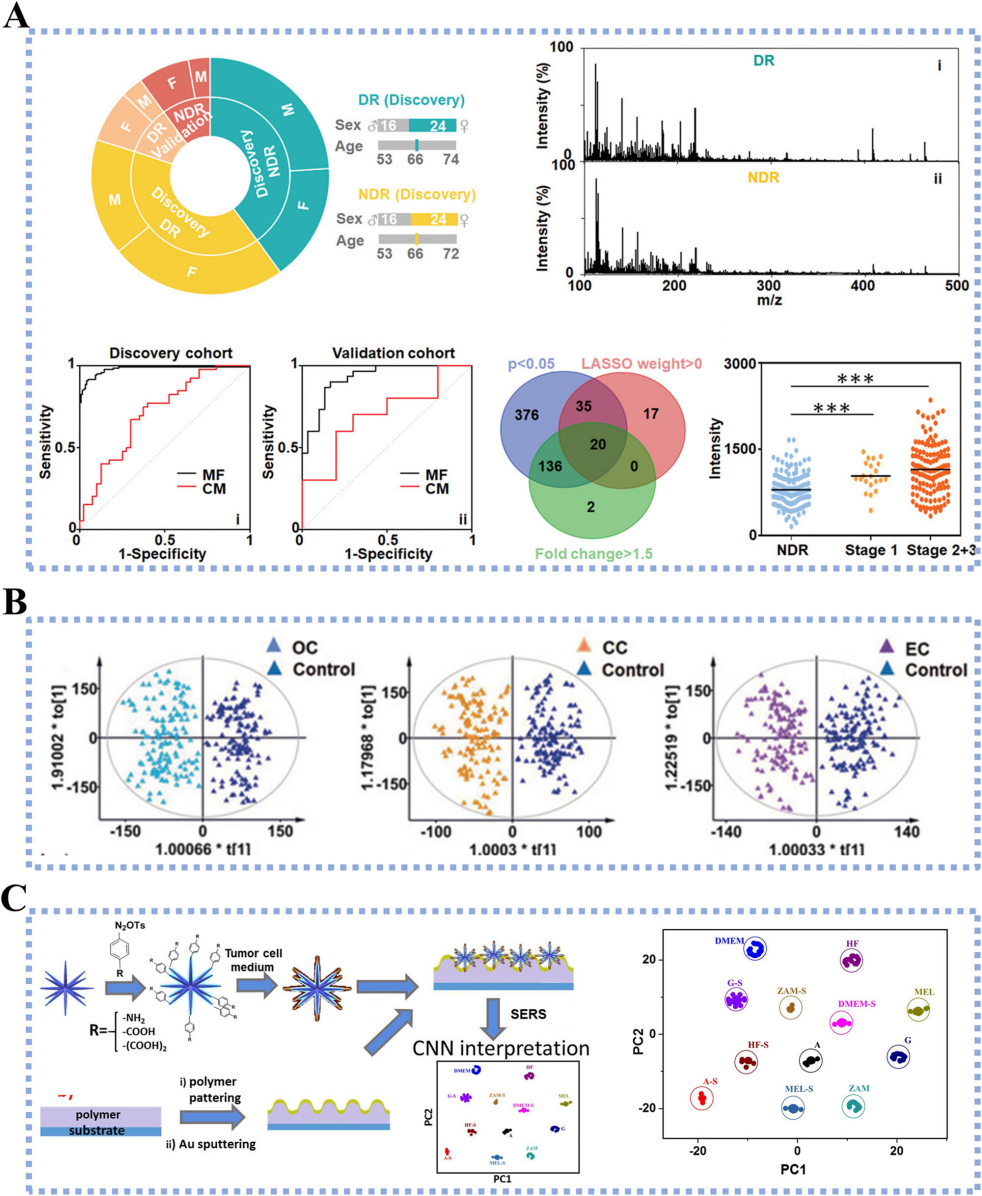
(A) Metabolic changes inspected by vanadium core‐shell nanorods fordiagnosing diabetic retinopathy. Particularly, vanadium core‐shell nanorods acted as matrices of LDI MS to improve the detection sensitivity of plasma metabolites. Reproduced with permission.^[^ ^109^ ^]^ Copyright 2020, Wiley‐VCH. (B) Data interpretation of serum metabolic fingerprints toward diagnosis of gynecological cancers. In particularly, the matrix applied for LDI MS detection in this work was FeOOH‐@ZIF‐8 composites. Reproduced with permission.^[^ ^111^ ^]^ Copyright 2020, Wiley VCH. (C) The functionalized Au multibranched NPs served as SERS substrate for metabolic analysis of cells and realized the identification of tumor cells from normal cells with an accuracy of 100%. Reproduced with permission.^[^ ^118^ ^]^ Copyright 2020, Elsevier

Particularly, blood samples including serum and plasma, have been well explored for the diagnosis of different diseases in recent years.^[^ ^89^, ^109^, ^110^, ^111^, ^112^, ^113^, ^114^ ^]^ Especially, Qian's group was dedicated to developing novel nanomaterials and their conjugation with LDI MS systems for the untargeted metabolic fingerprinting of blood samples.^[^ ^15^, ^89^, ^91^, ^109^, ^115^ ^]^ For example, Vedarethinam et al. extracted the plasma metabolic fingerprints using LDI MS, which was assisted by V_2_O_5_ nanorods for enhanced sensitivity.^[^ ^109^ ^]^ A total of 100 plasma samples from diabetic retinopathy (DR) and non‐DR control patients were analyzed and utilized for the construction of a diagnostic model with sensitivity/specificity of 94%/90% (AUC of 0.967) for discovery cohort and 90%/83% (AUC of 0.926) for an independent validation cohort (Figure 9A).^[^ ^109^ ^]^ Similarly, Pei et al. designed FeOOH@ZIF‐8 composites for metabolic analysis of serum samples.^[^ ^111^ ^]^ They analyzed the serum samples from 89 gynecological cancer patients and 80 healthy controls and extracted the corresponding metabolic fingerprints for diagnosis model building. The above diagnostic model yielded the accurate differentiation of three subtypes of gynecological cancer, as shown in Figure 9B. Based on blood analysis, the metabolic fingerprints acquired from nanomaterial‐assisted analytical platforms reach comparable performance to the clinical gold standard of biopsy and improve patient compliance due to the minimal invasiveness.

In parallel to blood samples, urine samples always contain higher concentrations of inorganic salts, placing strict requirements for salt endurance of nanomaterial‐based matrix. Yang et al. extended a polymer@Ag‐assisted LDI MS to the analysis of human urine samples and encoded kidney diseases based on the extracted metabolic fingerprints.^[^ ^116^ ^]^ In this case, metabolic fingerprints were collected from 80 healthy controls and 108 kidney disease patients, by consuming 1 µl of urine samples without any complex pretreatment process owing to the high salt tolerance of the nanomaterials. A diagnostic model was built for subtyping kidney diseases with an AUC of 0.86. Besides, the metabolic fingerprints based on other biofluid samples have also exhibited irreplaceable role in unveiling specific disease (e.g., CSF for cerebral disease and saliva for oral disease), which calls for more attention in metabolic related research.

Metabolic fingerprints can also be collected from other biospecimens, including but not limited to cells^[^ ^117^, ^118^, ^119^, ^120^ ^]^ and exosomes.^[^ ^121^, ^122^ ^]^ The metabolic fingerprints at cellular level are fundamentally significant for disease diagnosis and management.^[^ ^123^, ^124^ ^]^ Erzina et al. constructed a multibranched Au NPs with various chemical groups functionalized as the substrate to enhance the sensitivity of SERS detection. This platform was further applied for metabolic investigation of normal and cancer cells, achieving a classification accuracy of 100% between cancer cell and normal cell by incorporating with convolutional neural network algorithm (Figure 9C).^[^ ^118^ ^]^ Moreover, metabolic fingerprints at subcellular level also provide critical insights to encode disease. Sun et al. constructed a plasmonic gold chip and combined it with microarray for metabolic fingerprints analysis.^[^ ^121^ ^]^ They performed the metabolic fingerprinting of exosome with a volume of 500 nl and obtained a series of metabolite peaks, which could identify 10 lung cancer patients from 10 healthy controls by orthogonal partial least squares discriminant analysis (*R* ^2^ = 0.994, *Q* ^2^ = 0.716, *p* < 0.0001). The above results are promising to the clinical diagnosis of lung cancer but might suffer from unrepresentative sample size and lack of an independent validation cohort.

In general, the untargeted metabolic fingerprints offer a more comprehensive view with timely feedback of the human body. Further considering the aid of nanomaterials, metabolic fingerprinting will reach an advanced detection sensitivity and hold the promise to predict disease occurrence at very early stage.

## 4 CONCLUSIONS AND FUTURE PERSPECTIVES

Nanomaterials have found extensive applications in metabolic analysis, with enhanced selectivity and sensitivity. Here, we describe the functional roles and bio‐applications of nanomaterial‐based analytical approaches toward IVD applications.

The future directions of nanomaterials‐assisted metabolic analysis toward IVD can be illustrated regarding nanomaterial development, metabolic analysis, and related applications. For nanomaterial development, nanocomposites with anisotropic structures display some intriguing properties compared to regular isotropic structured particles, requiring the controllable preparation methods and optimization of each component to exploit their synergic effects. For metabolic analysis, a foundational future direction is to decipher the untargeted metabolic fingerprints by advanced algorithms, contributing to the new biomarker discovery and related encoding of disease mechanisms. For IVD applications, integrated assay devices and the corresponding detection kits wait for exploration with superior adaptability as well as efficient sensitivity and specificity in clinical scenarios.

In summary, nanomaterial‐based metabolic analysis is always accompanied by accurate quantitation and high signal throughput, which have important practical value in the fields such as disease diagnostics and personalized therapeutics. Further developments of nanomaterials‐based metabolic analysis will have far‐reaching implications to the research of precision medicine.

## CONFLICT OF INTEREST

The authors declare no conflict of interest.
